# Intracranial Atherosclerosis Versus Primary Angiitis of the Central Nervous System: a Case Report

**DOI:** 10.7759/cureus.3031

**Published:** 2018-07-23

**Authors:** Joshua A Ronen, Aileen Nguyen, Jerrica N Mueller, Hyonju Lee

**Affiliations:** 1 Internal Medicine, Texas Tech University Health Sciences Center of the Permian Basin, Odessa, USA; 2 Internal Medicine Resident Physician, St. Mary's Medical Center, San Francisco, USA; 3 Internal Medicine Resident Physician, St. Agnes Medical Center, Fresno, USA; 4 Ross University School of Medicine, California Hospital Medical Center, Los Angeles, USA

**Keywords:** reversible cerebral vasoconstriction disorder, primary angiitis of the central nervous system, cns vasculitis, vasculitis, intracranial atherosclerosis, cerebral angiography, magnetic resonance angiography

## Abstract

Primary angiitis of the central nervous system (PACNS) is a rare disease with various clinical presentations. It is the preferred name for vasculitis that is confined to the central nervous system (CNS) and is often considered a diagnosis of exclusion in vascular or inflammatory CNS diseases.

This case describes a 46-year-old right-handed female with a past medical history of hypertension (HTN), hyperlipidemia (HLD), diabetes mellitus type two (DM2), obesity, and hemorrhagic stroke who was transferred from an outside facility after a one-month hospitalization to be evaluated for CNS vasculitis. Emergency medical personnel who brought the patient to the receiving hospital endorsed dysarthria and fluctuating level of cognition. Before she was transferred, the patient underwent a series of computed tomography (CT) and magnetic resonance imaging and angiographies (MRI/MRA) as well as four-vessel angiography. The studies revealed multiple bilateral striatal and cortical infarcts, scattered narrowing and occlusion of major cerebral vasculature, as well as other signs initially more suggestive of intracranial atherosclerosis than CNS vasculitis. Before she was transferred, imaging demonstrated a new cortical stroke. Hypercoagulable studies were positive for protein C deficiency although ensuing echocardiograms with normal ejection fractions were negative for a source of cardioembolism. Having undergone extensive rheumatological, radiological, and neurosurgical evaluation in the receiving facility, recommendations were made for the patient to undergo leptomeningeal biopsy to confirm the diagnosis of vasculopathy and to specifically rule out intracranial atherosclerosis and reversible cerebral vasoconstriction syndrome (RCVS).

## Introduction

Primary angiitis of the central nervous system (PACNS), also known as central nervous system (CNS) vasculitis, was first described by Cravioto and Feigin in 1959 while dissecting brains post-mortem, as explained by Hajj-Ali et al. [1]. Today, PACNS is defined as a disease characterized by inflammation and destruction of blood vessels isolated to the CNS. This rare, but potentially debilitating, disease has an annual incidence rate of 2.4 cases per one million person-years with a two to one male predominance. Angiitis of the CNS is considered secondary when it occurs within the context of another disease process. Various etiological factors have been proposed in secondary angiitis including systemic inflammatory diseases, cerebral amyloid angiopathy, Varicella zoster virus, West Nile virus, mycoplasma gallisepticum, and human immunodeficiency virus (HIV). Although the exact etiology of PACNS is unknown, Hajj-Ali and Calabrese explain that the pathological process is best understood as an inflammation of the CNS that causes cerebral blood vessels to become narrowed, occluded, and thrombosed, resulting in tissue ischemia and necrosis [2].

## Case presentation

The patient is a 46-year-old right-handed female with a past medical history of hypertension (HTN), hyperlipidemia (HLD), diabetes mellitus type two (DM2), obesity, and hemorrhagic stroke who was transferred from an outside facility to be evaluated for CNS vasculitis. She was admitted to this outside facility for a four-week period prior to being transferred to the primary facility for further evaluation over a subsequent 23-day period. Total duration of hospitalization at both the facilities was close to 7.5 weeks. Approximately one week into the initial four-week admission, her family found that she was very lethargic with diminished responsiveness and pronounced difficulty speaking. In the emergency room (ER), her blood pressure was measured at 243/129 mmHg with a blood glucose value greater than 400 mg/dL. She was started on aggressive antihypertensive therapy and underwent a series of diagnostic tests. Dual antiplatelet therapy (DAPT) consisting of aspirin and clopidogrel was initiated in combination with high-dose atorvastatin. With respect to her lethargy and fluctuating cognition, there was concern that she may be experiencing complex partial seizures, so lacosamide was also started.

A baseline computed tomography (CT) scan of the head without contrast showed multiple indeterminate lacunar infarcts involving the head of the right caudate nucleus and left corona radiata. The same day, a magnetic resonance imaging (MRI) was performed and elicited similar findings with the addition of bilateral punctate infarcts of the left thalamus, right periventricular white matter, and right centrum semiovale. Magnetic resonance angiography (MRA) done on the following day showed high-grade stenosis of the left middle cerebral artery (MCA), in addition to markedly diminished caliber of the right MCA and high-grade stenosis involving the left posterior inferior cerebellar artery (PICA). Bilateral carotid ultrasounds showed very mild plaques. An angiogram exhibited an occluded left posterior cerebral artery (PCA) distally and was also suggestive of advanced intracranial atherosclerosis (more so than would be expected in CNS vasculitis). There was no evident change from day two to day six of this hospital course. A spinal tap performed at the end of the first week demonstrated elevated protein and IgG synthesis rate (16.4), which was concerning for CNS vasculitis. Appreciating the contrast between the imaging and spinal tap findings, CNS vasculitis could not be ruled out. The patient was started on intravenous (IV) corticosteroids briefly, however, the medication was discontinued due to worsening hyperglycemia that was progressively difficult to control. Near the end of the third week of hospitalization, a repeat MRI showed a new small stroke in the left subcortical parietal white matter.

The patient was transferred to the primary facility after this initial month of hospitalization, at which time the patient had a National Institutes of Health Stroke Score (NIHSS) of seven. She was alert and oriented to person only and able to follow simple commands. Significant findings on subsequent blood testing revealed leukocytosis (12.2), elevated absolute neutrophil count (ANC) at 11.3, hyperglycemia (314 mg/dL), HbA1c of 9.6%, mildly elevated erythrocyte sedimentation rate (ESR) at 36, positive herpes simplex virus type one (HSV1), and the presence of IgG and hepatitis B core antibody (HBcAb). Workup for hypercoagulable state was negative for Factor V Leiden and antithrombin deficiencies, though notably protein C was elevated. A repeat spinal tap on hospital day one showed elevated levels of protein (122), but also demonstrated an elevation in myelin basic protein (6.09). Otherwise, the patient was afebrile and hemodynamically stable on admission. On hospital day two, rheumatology was consulted. In order to confirm the suspected diagnosis of CNS vasculitis, the specialist recommended a leptomeningeal biopsy and IV corticosteroids in the interim. Although angiography is very sensitive, it is nonspecific as it cannot distinguish between vasculitis and reversible cerebral vasoconstriction syndrome (RCVS). Consequently, the angiography that the patient had undergone earlier in her hospital course could not provide us with a definitive diagnosis, thus warranting the biopsy of the brain.

A baseline transthoracic echocardiogram (TTE) obtained on hospital day three revealed a left ventricular ejection fraction (LVEF) of 57 +/-5 percent with mild dilation of the left atrial cavity. Repeat imaging showed much of the same findings, however, radiology recommended further workup for underlying CNS vasculitis. Over hospital day four to six, the working diagnosis was “multifocal bihemispheric strokes with no clear etiology with an encephalopathic process.” The patient’s cardiovascular risk factors continued to be treated and monitored (lipids, blood pressure, and sugars), and a more extensive rheumatological workup was ordered. Continuous electroencephalogram (EEG) monitoring on hospital day eight also showed evidence of diffuse encephalopathy although there were no epileptiform changes or seizures recorded. Over the second week of hospitalization, a new left cerebellar infarct was detected on MRI, at which time steroids were tapered down and a transesophageal echocardiogram (TEE) was ordered. The results of this echocardiogram were unchanged in comparison to the baseline TTE. There was no thrombus detected in the left atrium, ruling out cardioembolic etiology of the new stroke. The CT studies of the chest and abdomen were negative for any findings pertinent to the patient’s chief complaint.

During the third week of hospitalization, a repeat head CT without contrast revealed additional recent infarcts. A four-vessel angiogram showed 50% stenosis in the petrous and cavernous segments of the left internal carotid artery (ICA), a completely occluded M1 segment of the left MCA, and multiple alternating foci of narrowing within the M2 and M3 branches of the right MCA as well as the P2 and P3 branches of the PCA. In consideration of these findings and given the fact that there is a considerable overlap between the imaging appearance of vasculitis and atherosclerotic disease, neither diagnosis could be excluded. A second rheumatology consult recommended that a leptomeningeal biopsy be considered prior to starting cyclophosphamide, effectively ruling in CNS vasculitis versus ischemic stroke. The neurosurgery team agreed to conduct the biopsy of the meninges and brain. However, after discussing the details of the procedure with the patient's family, her family decided against her having the procedure due to the risks associated with brain surgery and the debilitating neurologic deficits already suffered by the patient.

## Discussion

Primary angiitis of the central nervous system (PACNS) is a rare disorder that is typically considered a diagnosis of exclusion after a comprehensive approach including a detailed history, clinical findings, laboratory investigations, and radiological findings has been performed. Due to its multifaceted clinical manifestations and nonspecific MRI findings, reaching the diagnosis of PACNS continues to be a challenge for many practitioners.

According to John and Hajj-Ali recent PACNS cohorts have experienced favorable outcomes when early diagnosis and prompt treatment were initiated [3]. Given that a speedy diagnosis and aggressive treatment help to avoid permanent neurological damage, it is imperative to be familiar with the many mimics of PACNS that may delay proper diagnosis. As per Hajj-Ali et al., the most common imitator is a group of disorders known as the reversible cerebral vasoconstriction syndromes (RCVS) which includes: migrainous vasospasm, Call-Fleming syndrome, thunderclap headache associated with vasospasm, drug-induced cerebral arteritis, postpartum cerebral angiopathy, benign angiopathy of the CNS, and CNS pseudovasculitis [2]. Other conditions that should be considered in the differential, as Noufal and Schmidley and Suri et al. describe, include but are not limited to: atrial myxoma with embolization to the brain, secondary vasculitides, collagen vascular disorders, viral or bacterial infections, neoplasms, hypercoagulable states, and substance abuse [4-5].  

With respect to clinical presentation, Singhal et al., Bhattacharyya and Berkowitz and Lucke and Hajj-Ali believe that the majority of patients that are eventually diagnosed with PACNS complain of a thunderclap-type headache that progressively worsens over the course of a few weeks [6-8]. de Boysson and Pagnoux point out that patients can also present with a variety of neurological deficits, seizures, or more rarely, medullary or brainstem involvement requiring mechanical ventilation [9]. Interestingly, our patient did not present with any complaint of headache. Her lethargy, inability to speak, initial NIHSS score of 7 upon presentation, however, are in line with other frequent presentations of PACNS. According to Lucke and Hajj-Ali encephalopathy and cognitive dysfunction are among some of the most common signs [8]. Our patient exemplified the cognitive dysfunction as well as the encephalopathy which was evidenced on EEG.

As per Suri et al., definite diagnostic criteria proposed by Callabrese and Mallek in 1988 can be used to make the diagnosis of PACNS if all of the following criteria are satisfied:

1. History of unexplained neurological deficit that remains after vigorous diagnostic workup including lumbar puncture and neuroimaging studies.

2. Either classic angiographic evidence of vasculitis or histopathological evidence of vasculitis within CNS.

3. No evidence of systemic vasculitis or any other condition to which the angiographic or pathologic evidence can be attributed [5].

In our patient, the initial workup included a lumbar puncture with CSF analysis. As per Suri et al., CSF studies in PACNS patients often show modest lymphocytic pleocytosis and raised protein levels [5]. In line with a diagnosis of PACNS, the patient’s CSF smear showed no organisms but had an elevated protein level of 122, and the culture was negative for any growth.    

**Figure 1:**
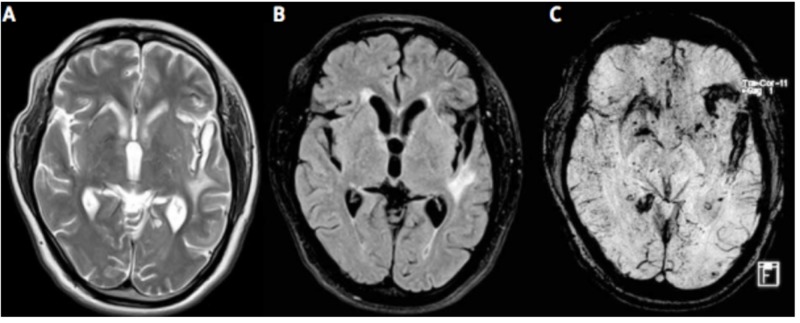
T2-weighted MR (A), FLAIR (B) and susceptibility weighted images (C) showing bilateral supratentorial white matter lesions, along with micro- and macro-hemorrhages. Ref. [5] This figure was used from Folia Neuropathologica with consent.

**Figure 2:**
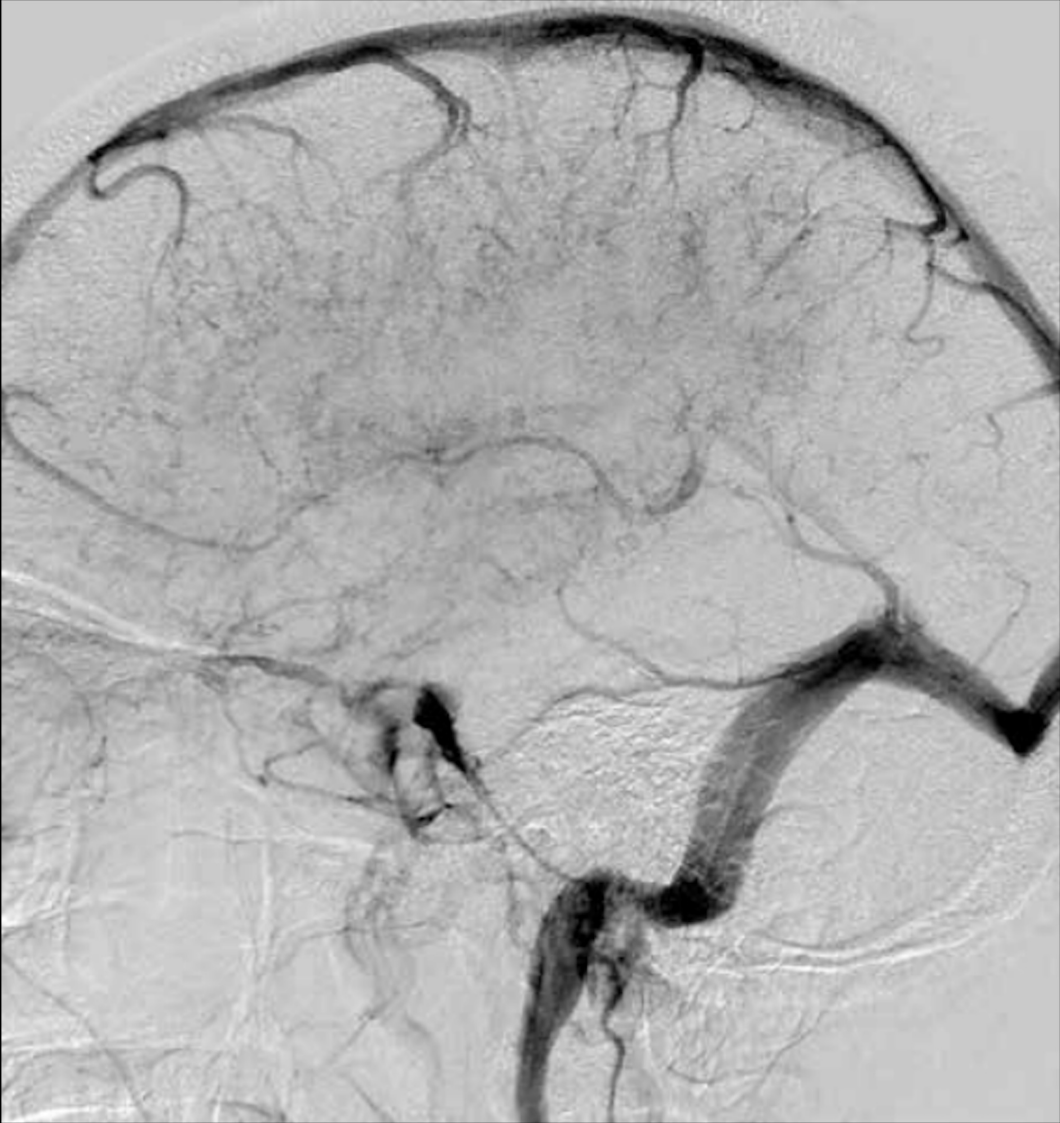
Venous phase of left internal carotid artery digital subtraction angiogram shows irregularities and indistinct pattern of deep veins suggestive of vasculitis. Ref. [5] This figure was used from Folia Neuropathologica with consent.

Neuroimaging in PACNS can be a useful tool, but is not without its limitations. Al Share et al. justify that MRI or MRA imaging is preferred to CT because they are more sensitive at detecting any type of vascular change [10]. Multiple MRI scans often show accumulating bilateral lesions over time that are located in the cortex, deep white matter or leptomeninges, as seen in Figure 1 [5]. An MRA will typically show evidence of irregular narrowing and beading of affected vessels [10]. In our patient’s case, four separate MRIs over a period of one month all showed evidence of multiple recurrent ischemic strokes affecting numerous vascular territories, a classic presentation of PACNS as Singhal et al., Lucke and Hajj-Ali and Noh et al. each describe [6, 8, 11]. The initial MRI showed evidence of punctate acute infarctions in the right caudate, left thalamus, right periventricular white matter, left PICA, and right centrum semiovale. The subsequent two MRIs demonstrated new small strokes in small vessels of the left cerebellum, new strokes in the subcortical parietal white matter, and three acute watershed infarcts of the right frontal corona radiata. The final MRI showed evidence of new infarctions in the left cerebellum (despite treatment with aspirin and plavix), with an appearance consistent with vasculitis. Angiograms can also be used when the clinical suspicion for PACNS is high, and when positive for vasculitis, demonstrate the characteristic irregular stenosis and dilation of various sized blood vessels (Figure 2) [5]. Suri et al. delineate that although angiograms in PACNS show blood vessel irregularities, as a diagnostic tool, it is neither specific (specificity as low as 30%) nor sensitive for the disease [5]. Further, angiography may even be normal in vasculitis that is limited to small vessels below resolution of conventional angiography. While diagnostic scans in our patient were positive, it is important for the practitioner to consider the timing of the scan within the course disease as PACNS has a progressive nature and early imaging could potentially provide negative results.

**Figure 3:**
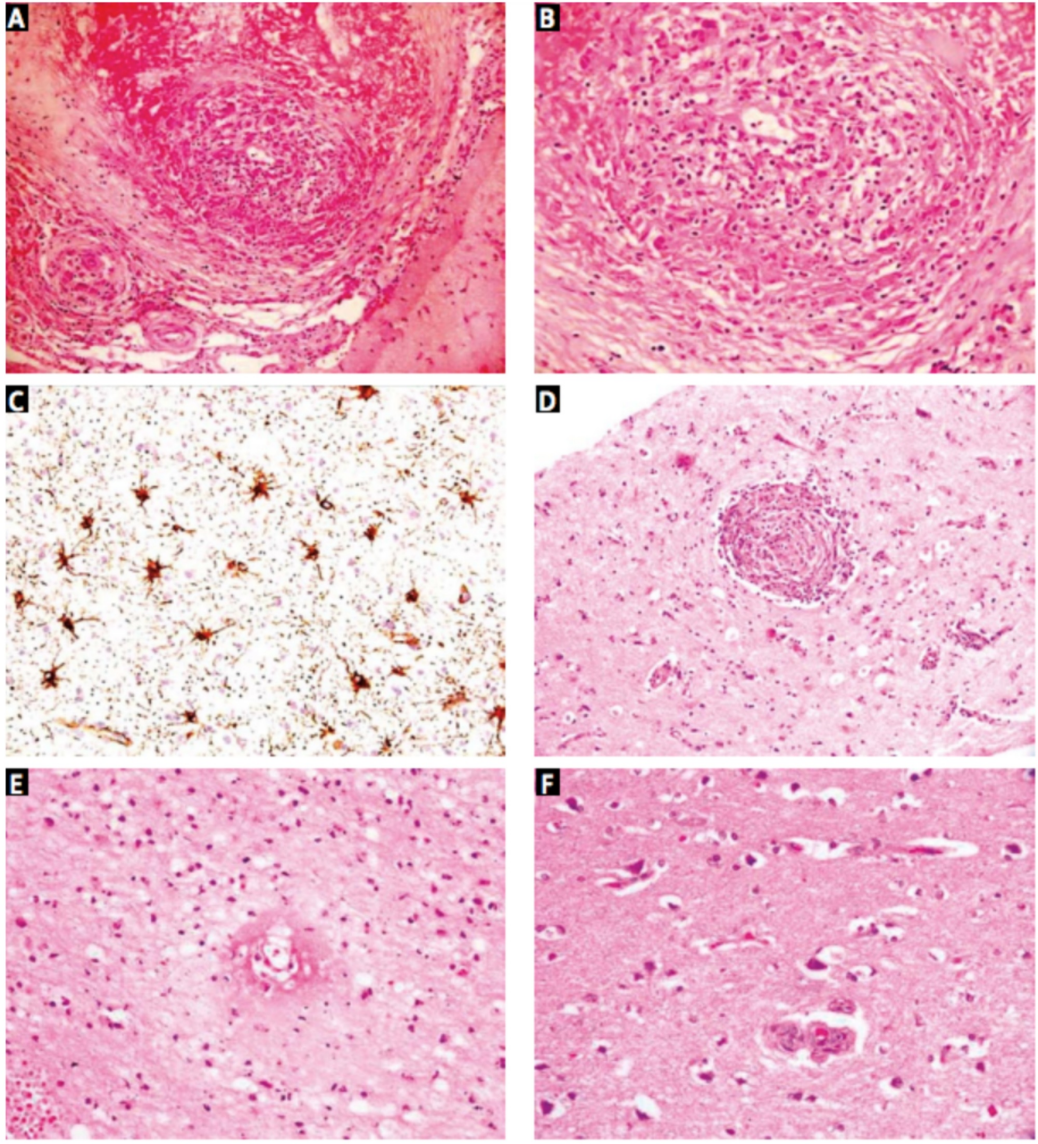
Photomicrographs showing (A) transmural granulomatous inflammation involving small and medium-sized leptomeningeal arteries (H&E, 200×). High magnification view (B) shows lymphocytes, epithelioid histiocytes, and giant cells (H&E, 400×). GFAP stain (C) highlights reactive astrocytosis (IHC, 400×). Similar granulomatous vasculitis seen in a small cortical artery (D; H&E, 200×) in case 6, along with transmural fibrinoid necrosis (E) in an adjacent cortical arteriole (H&E, 400×). Surrounding cortex (F) shows ischemic neurons (H&E, 400×). Ref. [5] This figure was used from Folia Neuropathologica with consent.

**Figure 4:**
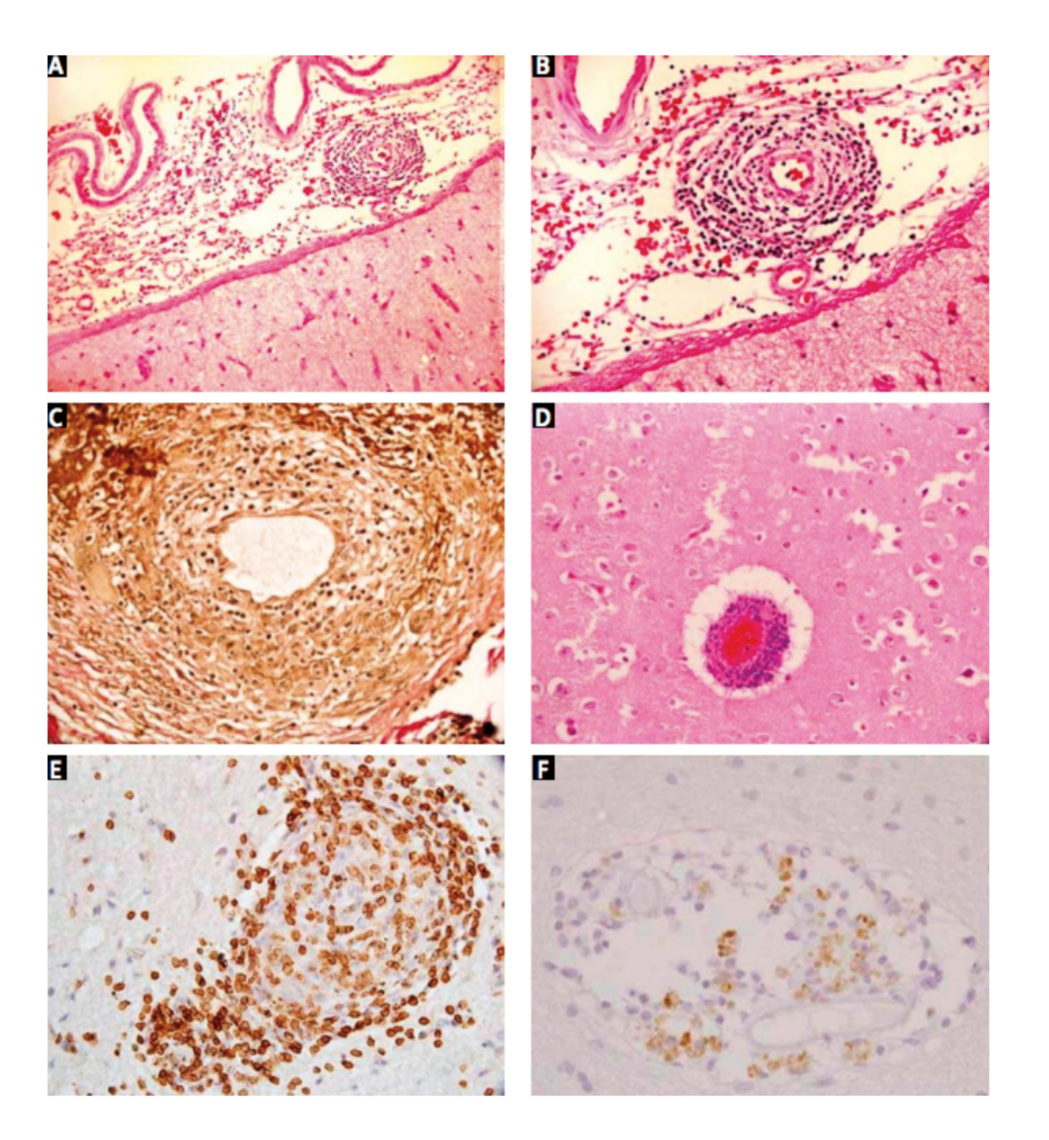
Photomicrographs showing (A) lymphocytic infiltrates within the wall of a leptomeningeal artery; there is no significant inflammatory infiltrate in the adjacent cortex (H&E, 100×). High power view (B) showing complete destruction of the vessel wall (H&E, 200×). VVG stain (C) demonstrates loss of internal elastic lamina (VVG, 400×). Cortical blood vessels (D) showed similar features (H&E, 200×). The inflammatory cells are predominantly CD3+ T cells (E) with only few CD20+ B cells (F) (IHC, 400×). Ref. [5] This figure was used from Folia Neuropathologica with consent.

Brain biopsy remains the gold standard for the diagnosis of PACNS. Suri et al. and Niu et al. say that the histology is reflective of the inflammatory nature of the disease: lymphocytes, plasmatocytes monocytes, and giant cells can be visualized in the acute phase, while astrocytes, multinucleated giant cells, and more rarely necrosis, are witnessed in the chronic phase (Figures 3 and 4) [5, 12]. Though brain biopsy remains the best available diagnostic test, it is highly invasive and Al Share et al. assert that up to as many as 25% of brain biopsies can be falsely negative [10]. This likely occurs secondary to either sampling error as the disease attacks in “patches,” or because affected vessels may not extend into the parenchyma or leptomeninges, therefore, a negative biopsy result does not effectively rule out PACNS as Al Share et al. and Niu et al. elaborate [10, 12]. Considering the challenging nature of diagnosing PACNS coupled with the high percentage of falsely negative biopsies, some cases are unfortunately not diagnosed until the patient succumbs to the neurological damage and post-mortem biopsies show evidence of PACNS. In our patient’s case, the first and only biopsy result demonstrated strong evidence for vasculitis, and the diagnosis of PACNS was made.

The case for prompt and proper diagnosis of PACNS cannot be overstated as treatment is imperative to preventing further neurological damage. Cravioto and Feigin explain that the current mainstay of treatment for PACNS is a high-dose steroid in combination with an immunosuppressant [1]. To emphasize the importance of early treatment, a case in the literature recounted by Sun et al. describes a 42-year-old man who was eventually diagnosed with PACNS, but whose initial diagnosis was a malignant glioma based on MRI findings [13]. He was placed on high-dose steroids for symptom control, but after a lack of improvement, underwent resection. The pathology report confirmed the presence of cerebral vasculitis. On an MRI scan of the brain one year later, the patient was noted to have a new lesion on the opposite side. At this time, the patient was started on a combination of methylprednisolone and cyclophosphamide, which resulted in a pronounced improvement of his condition. The patient was without relapse at the one year follow-up. This case demonstrates the importance of shrewd detection of common mimics of PACNS, and the necessity of prompt diagnosis and appropriate treatment in order to avoid unnecessary procedures and improve patient outcomes. Our patient, like the one mentioned above, was also placed on high-dose steroids (solumedrol) alone, which failed to alleviate her symptoms. Diagnostically speaking, this finding should point clinicians in the direction of PACNS.

Given that PACNS has variable clinical presentations along with improved prognosis with early diagnosis, physicians will benefit from being more knowledgeable of various aspects of the disease. These include being familiar with PACNS mimics, knowing which diagnostic studies yield the most accurate results, understanding that biopsies can be falsely negative, and that even though a patient does not present with some of the most common symptoms of PACNS, it does not necessarily mean they do not have PACNS.

## Conclusions

Prompt diagnosis and treatment initiation are imperative for achieving favorable outcomes in PACNS. With consideration of the common and uncommon variety of clinical manifestations, it is important for clinicians to recognize the many mimics of PACNS and be fully aware of the accuracy of the various imaging and laboratory studies available to aid in its diagnosis.
